# Identifying metabolic shifts in Crohn's disease using 'omics-driven contextualized computational metabolic network models

**DOI:** 10.1038/s41598-022-26816-5

**Published:** 2023-01-05

**Authors:** Philip Fernandes, Yash Sharma, Fatima Zulqarnain, Brooklyn McGrew, Aman Shrivastava, Lubaina Ehsan, Dawson Payne, Lillian Dillard, Deborah Powers, Isabelle Aldridge, Jason Matthews, Subra Kugathasan, Facundo M. Fernández, David Gaul, Jason A. Papin, Sana Syed

**Affiliations:** 1 Department of Pediatrics, Division of Gastroenterology, Hepatology & Nutrition, University of Virginia School of Medicine & UVA Child Health Research Center, University of Virginia, MR-4 Bldg, 409 Lane Rd., Charlottesville, VA 22908 USA; 2 Division of Pediatric Gastroenterology, Emory Children’s Center, Atlanta, GA USA; 3 Petit Institute for Bioengineering and Bioscience, Georgia Institute of Technology, Atlanta, GA USA; 4 School of Chemistry and Biochemistry, Georgia Institute of Technology, Atlanta, GA USA

**Keywords:** Biotechnology, Computational biology and bioinformatics, Biomarkers, Gastroenterology, Medical research, Biomedical engineering

## Abstract

Crohn's disease (CD) is a chronic inflammatory disease of the gastrointestinal tract. A clear gap in our existing CD diagnostics and current disease management approaches is the lack of highly specific biomarkers that can be used to streamline or personalize disease management. Comprehensive profiling of metabolites holds promise; however, these high-dimensional profiles need to be reduced to have relevance in the context of CD. Machine learning approaches are optimally suited to bridge this gap in knowledge by contextualizing the metabolic alterations in CD using genome-scale metabolic network reconstructions. Our work presents a framework for studying altered metabolic reactions between patients with CD and controls using publicly available transcriptomic data and existing gene-driven metabolic network reconstructions. Additionally, we apply the same methods to patient-derived ileal enteroids to explore the utility of using this experimental in vitro platform for studying CD. Furthermore, we have piloted an untargeted metabolomics approach as a proof-of-concept validation strategy in human ileal mucosal tissue. These findings suggest that in silico metabolic modeling can potentially identify pathways of clinical relevance in CD, paving the way for the future discovery of novel diagnostic biomarkers and therapeutic targets.

## Introduction

Crohn's disease (CD) is a chronic, relapsing inflammatory disease of the gastrointestinal tract. CD prevalence ranges between 96 and 318 per 100,000 person-years in North America alone, with pediatric patients being the fastest-growing CD incidence group^1–3^. Pediatric-onset CD affects the upper gastrointestinal tract more frequently than adult-onset disease and is more likely to follow a more severe disease course^4,5^. The progressive inflammation resulting from CD disrupts critical developmental periods in children, impeding physical and psychosocial growth^6^. Our current understanding of CD pathogenesis has expanded in recent years to include dietary and lifestyle factors, environmental influences, genetic predilection, and gut microbiome composition^7,8^. The initial clinical diagnostic evaluation of CD includes, but is not limited to, panels of blood and stool biomarkers, such as hemoglobin, C-reactive protein (CRP), erythrocyte sedimentation rate (ESR), albumin, and fecal calprotectin. These biomarkers have sensitivities ranging from 80 to 90% but relatively low pooled specificity to CD in pediatric patients^9,10^. For a definitive diagnosis, clinicians use invasive and often costly investigations such as endoscopy, biopsy, and small-bowel cross-sectional imaging^2^. Thus, there is a clear gap in highly specific biomarkers for CD diagnosis and subsequent prognosis.

The management protocols for CD hinge on achieving suppression of intestinal inflammation during an induction phase with subsequent maintenance of remission. Strategies for induction include a bottom-up or top-down approach using a combination of steroids, immunomodulators (azathioprine or 6-mercaptopurine), antibiotics, or biologics that inhibit tumor necrosis factor-alpha, such as infliximab and adalimumab^11–13^. Unfortunately, these medications have variable efficacy between patients and may cause severe side effects^14,15^. Therefore, clarifying pathways contributing to CD may reveal novel targets for directed therapy, reducing immunosuppression-related morbidity.

Constraint-based metabolic modeling has emerged as a useful in silico computational approach to investigate variations in the metabolism under certain biological conditions by analyzing large-scale relationships between genotypes and phenotypes^16^. Through the interrogation of transcriptomic variations in metabolism, we may be able to illuminate not only pathophysiological pathways for targeted therapeutics but also specific metabolic biomarkers of CD. Genome-scale metabolic Network Reconstructions (GENREs) provide a platform to study transcriptomic data in the context of metabolic shifts between various disease states^17^. Recon3D is the most comprehensive human metabolic network reconstruction to date; it includes gene, protein structure, and metabolite data from over 3,500 genes^18^. Genes related to metabolism are annotated and matched to the enzymes they code for and the reactions they catalyze to generate a catalog of gene-protein-reaction (GPR) associations. Flux Balance Analysis (FBA) is one method that can be employed to create a mathematical function of the flux of substrates along a given pathway in a metabolic network reconstruction with the assumption of a steady state^19^. FBA data obtained from constrained-based modeling can subsequently be used as inputs for machine learning models to perform analysis in a sample-specific manner to identify relevant targets for biotechnology and biomedical interventions. FBA-based methodologies effectively offer an approach to pruning unused pathways to identify relevant utilized pathways from the dense reaction-centric metabolic network. Using one pruning approach, parsimonious flux balance analysis (pFBA), metabolic reactions relevant to specific physiological or pathological conditions can be determined by identifying the most efficient reactions in generating biomass^20–23^. Reaction Inclusion by Parsimony and Transcript Distribution (RIPTiDe) provides an additional method for pruning reactions by factoring in transcriptomic abundances to identify the most important energy-efficient metabolic pathways in a given biological state. In CD, this multi-tiered in silico approach potentially allows us to discern the metabolic underpinnings of CD and clarify possible biomarkers and druggable targets^24–26^.

To this end, we have used an FBA-based machine learning (ML) framework for studying altered metabolic reactions between patients with CD and controls, leveraging 'omics data from a public repository from the Risk Stratification and Identification of Immunogenetic and Microbial Markers of Rapid Disease Progression in Children with Crohn's Disease (RISK) study, one of the largest pediatric inception cohorts of children with a new diagnosis of CD, to identify biomarkers found in the blood or stool to help predict which children are at risk of developing complications^1,27^. We aimed to (1) identify a list of metabolic pathways that have altered flux or flow in CD versus controls using in silico metabolic modeling utilizing ileal transcriptomics (2) explore the overlap of metabolic pathways in CD ileal enteroids, and (3) pilot an approach to validate our findings of altered metabolism using untargeted mass-spectrometry-based metabolomics in ileal mucosal biopsies. We hypothesized that the predicted metabolic shifts in the CD ileum will mirror those in the enteroid model, as well as those metabolites identified using metabolomics, and thus this metabolic modeling approach potentially holds power to elucidate novel biomarkers and therapeutic targets, allowing for improved methods for the diagnosis and treatment of Crohn's disease.

## Results

### Demographics of clinical cohorts RISK and enteroids

The RISK prospective pediatric inception study included n = 243 patients with a new diagnosis of Crohn's disease and n = 43 age-matched control patients. Our analysis included n = 163 patients (59% male) diagnosed with CD localized to the ileum with a mean (± SD) age of 11.9 (± 3.1) years and n = 42 controls (62% male) with a mean (± SD) age of 11.1 (± 3.1) years. In addition, publicly available RNA sequencing data from enteroids that were derived from a previously established cohort of n = 16 patients with CD with a mean age (± SD) of 15.8 (± 3.2) years, 88% of whom were male, and n = 12 non-IBD controls with a mean (± SD) age of 11.4 (± 3.5), 42% of whom were male was also used for this analysis^28^. A descriptive summary of both cohorts is outlined in Table 1.

**Table 1:** Demographic data of cohorts used for metabolic modeling: A. RISK cohort. Publicly available Risk Stratification and Identification of Immunogenetic and Microbial Markers of Rapid Disease Progression in Children with Crohn’s Disease (RISK) cohort data was accessed using GEO accession series GSE57945. RISK is a prospective inception cohort study that enrolled 1,276 pediatric patients with IBD diagnosed at 28 sites in North America between 2008 and 2012. It includes n = 163 patients with ileal Crohn's disease and n = 42 healthy controls. B. Enteroid cohort. Gene expression data for all mucosal terminal ileal biopsies used to generate enteroids included in this study have been accessed from the Sequence Read Archive (SRA) using the SRA series accession SUB7687325. *Age at diagnosis; SD: standard deviation.

	A. RISK Cohort		B. Enteroid Cohort	
	Crohn’s disease n = 163	Controls n = 42	Crohn’s disease n = 16	Controls n = 12
Age* (yr), mean (SD)	11.9 (3.1)	11.1 (3.1)	15.8 (3.2)	11.4 (3.5)
Male (%)	59% (n = 96)	62% (n = 26)	88% (n = 14)	42% (n = 5)

**Figure 1:**
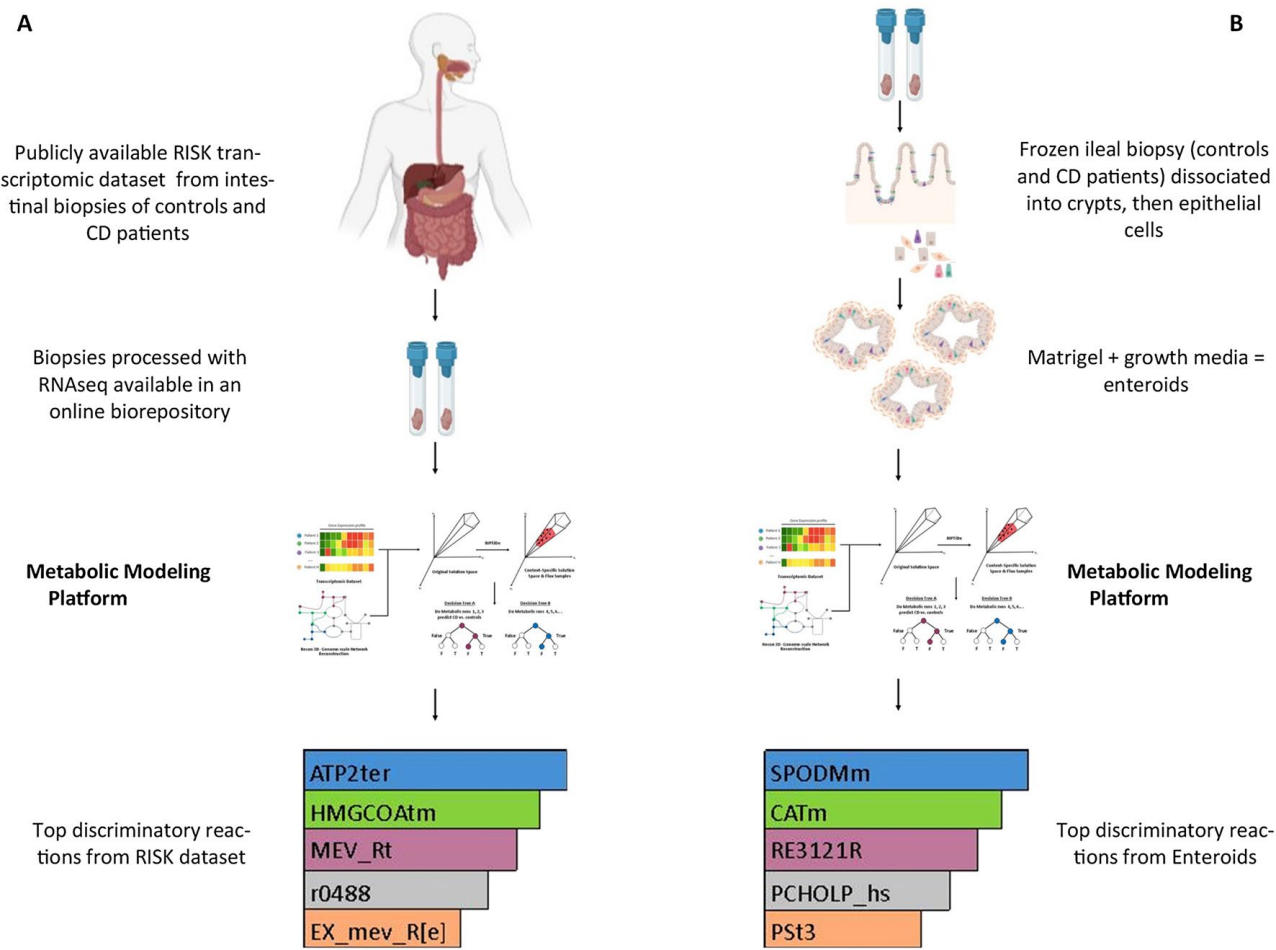
Schematic Overview of the performed metabolic modeling experiments: (**A**) On publicly-available RISK transcriptomic dataset and (**B**) Enteroid cultures that were generated for CD pediatric patients and controls.

To identify peripheral biomarkers and metabolic pathways indicative of CD, we used a computational metabolic modeling approach to generate metabolic profiles of the disease (Fig. 1). We independently constructed the metabolic profiles and lists of relevant reactions for CD and control groups from (1) the open access RISK transcriptomic dataset with Recon3D and (2) the enteroid transcriptomic dataset with Recon3D, as shown in Fig. 1.

### Differentially utilized reactions in the RISK metabolic model

**Figure 2:**
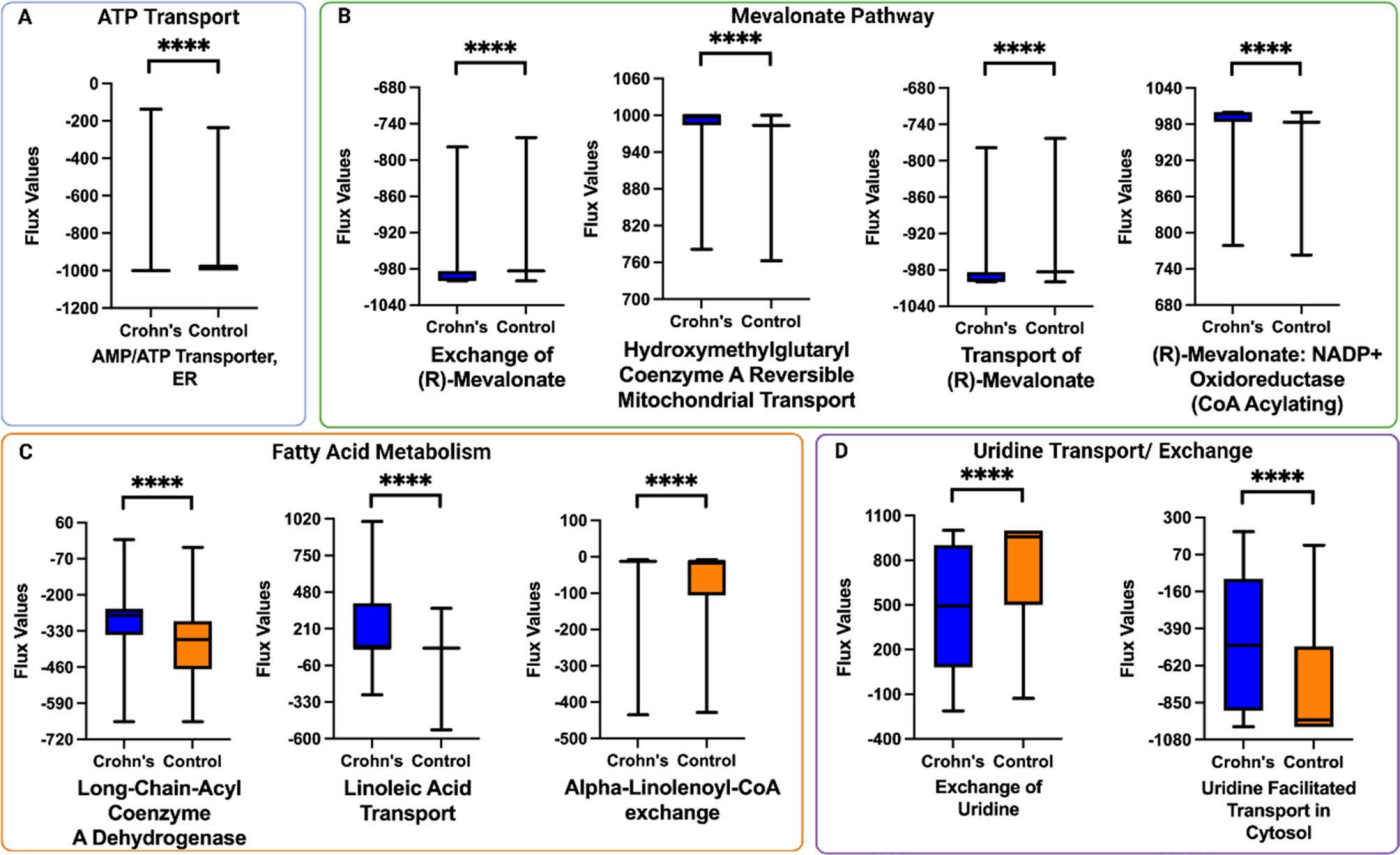
Metabolic pathways with an altered flow in patients with Crohn's disease (CD) versus control patients. The Flux Balance Analysis/Random Forest framework outlined in Fig. 1 were applied to the RISK dataset. After obtaining the list of top metabolic reactions that were altered between CD and control groups, these reactions were grouped into "families" (**A–D**) based on the biological processes they were involved in: (**A**) ATP transport processes were found to have significant rates of flux. (**B**) Mevalonate pathway. Several enzymes within the mevalonate pathway were altered in patients with CD, including the exchange of R- mevalonate, hydroxymethylglutaryl coenzyme A reversible mitochondrial transport, transport of R- mevalonate, and R- mevalonate NADP + oxidoreductase (CoA acylating), (**C**) Fatty acid oxidation. Long-chain-acyl Coenzyme A dehydrogenase, linoleic acid transport, and alpha-linolenoyl-CoA exchange (**D**) Uridine transport and exchange. For all graphs, the x-axis describes the reaction that is altered between controls (orange) and diseased states (blue). The y-axis shows the flux values generated by RIPTiDe by analyzing the flow of metabolites through an ileal-specific metabolic network reconstruction. The scale of flux values (y-axis) varies with the reactions as the efficiency of different metabolic pathways in generating biomass varies in a given biological system. A Mann–Whitney U test was done to compare reactions that varied between patients with Crohn's disease and controls. The stars (*) are used to flag levels of significance. *p < 0.05, **p < 0.01, ***p < 0.001. AMP: adenosine monophosphate; ATP: adenosine triphosphate; ER: endoplasmic reticulum; NADP + : nicotinamide adenine dinucleotide phosphate CoA: Coenzyme A.

Next, we sought to develop a metabolic model of ileal tissue from patients with CD. To this end, we applied RIPTiDe to publicly available transcriptomic data from the previously completed RISK study^1^. We identified context-specific metabolic pathway activity in both CD and control groups (n = 166, n = 42, respectively). This metabolic profiling revealed that the activity of approximately 200 reactions varied between control and CD populations (Supplemental Table 1), demonstrating that CD is associated with significant changes in ileal metabolism. Subsequently, we sought to determine whether this metabolic modeling approach could potentially identify CD using the metabolic signature generated from a transcriptomic validation dataset. Using random forest classification to identify discriminating reactions, our models determined whether a sample was from diseased versus control tissue, achieving 80% accuracy after 100 train-test splits in classifying CD versus control patients. Given these high accuracy measures, we can confidently use these methodologies to identify the most relevant metabolic alterations specific to CD. The average number of reactions pruned from the RISK dataset was 314 reactions (Supplemental Table 3). We next identified the top metabolic reactions that differentiated disease from controls. The reaction IDs, as documented in the Recon3D human network model were: AMP/ATP Transporter, endoplasmic reticulum (ATP2ter), hydroxymethylglutaryl coenzyme A reversible mitochondrial transport (HMGCOAtm), transport of (R)-mevalonate (MEV_Rt), (R)-mevalonate: NADP + oxidoreductase (CoA Acylating) (r0488), exchange of (R)-mevalonate (EX_mev_R[e]), alpha-linolenoyl-CoA metabolism (sink_lnlncacoa[c]), exchange of uridine (EX_uri[e]), long-chain-acyl Coenzyme A dehydrogenase (r1466), linoleic acid metabolism (sink_lnlc[c]), and uridine facilitated transport in the cytosol (URIt) (*p* value < 0.001; Supplemental Table 1). These top reactions were further grouped based on the metabolites involved in the reactions and the overall biological process. Upon literature review, the reactions were grouped under the gross biochemical pathways to which they belonged. The reactions found to be significantly altered were the mevalonate pathway, fatty acid oxidation, and uridine transport (*p* value < 0.001; Fig. 2A–D). Our model identified several alterations in the mevalonate pathway, including the conversion of mevalonate to HMG-CoA and HMG-CoA transport and exchange (Fig. 2B), and in the fatty acid oxidation pathway, such as linoleic acid transport, alpha-linolenoyl CoA exchange, and reactions involving long-chain-acyl coenzyme A dehydrogenase (Fig. 2C). Finally, alterations noted in uridine transport included the exchange of uridine and uridine-facilitated transport in the cytosol (Fig. 2D). Together, these findings highlight the significant metabolic differences between controls and patients with CD.

### Differentially utilized reactions in the enteroid metabolic model

Enteroids are a valuable tool in modern research, facilitating the ex vivo manipulation and investigation of human tissues. RNA sequencing (RNA-seq) from recent studies has demonstrated that enteroids derived from patients with CD have a 90% similarity in protein-coding genes compared to intestinal crypts isolated from these patients^28^. Furthermore, isolated enteroids were found to have similar cytokine and growth factor profiles as inflamed epithelium in patients, suggesting that enteroids exhibit similar characteristics to the diseased epithelium^28^. Therefore, we hypothesized that enteroid transcriptomes could be used to create a generalized in vitro metabolic model for gastrointestinal disease to distinguish quantifiable and targetable metabolic pathways. To test this hypothesis, we used our analytical pipeline designed for the RISK dataset outlined above on enteroid RNA-seq data from a CD cohort^28^. Enteroids were generated as previously reported, and RNA extracted for downstream analysis was accessed from the National Library of Medicine^28^. RNA sequencing data was RPKM normalized and used for downstream metabolic modeling to validate our in silico approach in a tissue CD model. Subsequently, we applied RIPTiDe, followed by random forest, to this enteroid transcriptomic dataset and extracted metabolic pathways most energetically productive to the CD model, as detailed in the methods section. Of note, our model had an average accuracy of 56% in classifying whether transcriptomic data originated from either CD or control patients within this validation cohort of enteroids after 100 train-test validation splits. Even though our accuracies were on average low when classifying patients with CD versus controls, we hypothesized that these data could potentially reveal significant alterations in the metabolic profiles of CD patients compared to control patients. Thus, as detailed in our methods section, we studied the top relevant reactions from only those train-validation splits where our detection accuracy was high enough (> 70%) to ensure the discriminability between CD and control patients to demonstrate the promise of enteroid models to serve as in vitro models of disease. The number of reactions in pruned in the enteroid dataset was 651 (Supplemental Table 3).Together, these data reveal significant alterations in the metabolic profiles of CD patients compared to control patients.

**Figure 3:**
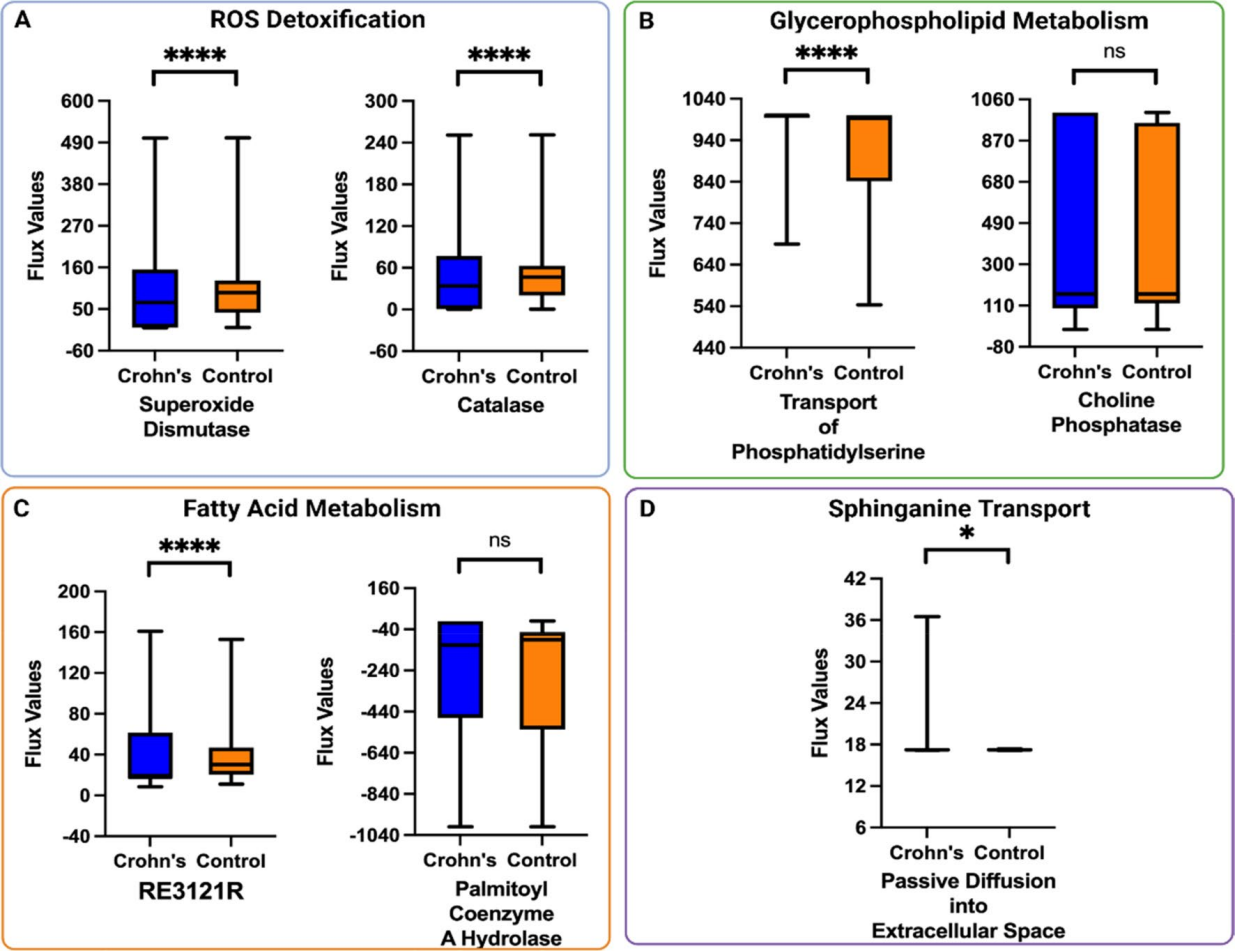
Metabolic pathways with an altered flow in enteroids generated from patients with Crohn's disease (CD) versus control patients: Our Flux Balance Analysis/Random Forest framework was applied to RNA sequencing data from enteroid models generated from n = 16 patients with Crohn's disease and n = 12 healthy controls. After obtaining the list of top metabolic reactions that were altered between the CD and control groups, these reactions were grouped into "families" (**A–D**) based on the biological processes they were involved in. (**A**) ROS detoxification: superoxide dismutase and catalase, (**B**) Glycerophospholipid metabolism: choline phosphatase and the transport of phosphatidylserine. (**C**) Fatty acid oxidation: RE3121R is involved in pentaenoyl coenzyme A metabolism and palmitoyl coenzyme A hydrolase (**D**) Sphinganine transport. For all graphs, the x-axis describes the reaction that is altered between controls (orange) and diseased states (blue). The y-axis shows the flux values generated by RIPTiDe by analyzing the flow of metabolites through an ileal-specific metabolic network reconstruction. The scale of flux values (y-axis) varies with the reactions as the efficiency of different metabolic pathways in generating biomass varies in a given biological system. A Mann–Whitney U test was done to compare reactions that were varied between patients with Crohn's disease and controls. The stars (*) are used to flag levels of significance. *p < 0.05, **p < 0.01, ***p < 0.001, ns: not significant. ROS: reactive oxygen species.

Following metabolic modeling of these enteroids derived from CD patients, we used the Recon3D database to categorize the reaction identification (IDs) of the most energy-efficient reactions in the small bowel. These reaction IDs were superoxide dismutase (SPODMm), catalase (CATm), RE3121R, a reaction involved in fatty acid oxidation, choline phosphatase (PCHOLP_hs), transport of phosphatidylserine (PSt3), passive diffusion of sphinganine into extracellular space (SPHGNte), palmitoyl coenzyme A hydrolase (RE0577C), exchange of coproporphyrinogen (EX_HC01610[e]), and HMR_0692, and a biomass reaction (biomass_reaction), two reactions involved in energy exchange (Fig. 3, Supplemental Table 1).

The metabolic reactions in the enteroids were classified using the subsystems given on the Virtual Metabolic Network and literature review as belonging to six metabolic subsystems: reactive oxygen species (ROS) detoxification, fatty acid oxidation, glycerophospholipid metabolism, fatty acid synthesis, transport, and exchange/demand^18,29,30^. These were further grouped based on the metabolites involved in the reactions and overall biological process, revealing four subgroups: ROS detoxification, fatty acid metabolism, glycerophospholipid metabolism, and sphinganine transport (Fig. 3A–D). Alterations to the ROS detoxification reactions included superoxide dismutase and catalase. These enzymes occur in series to detoxify superoxide and then catalyze the decomposition of hydrogen peroxide into oxygen and water. Alterations in glycerophospholipid metabolism included phosphatidylserine transport and phospholipase D metabolism. Phosphatidylserine is a lipid component of cellular membranes and has non-structural roles in cell signaling and apoptosis. Phospholipase D serves as a key component of multiple signaling and metabolic pathways. These findings suggest that CD is associated with alterations in several diverse metabolic pathways.

### Comparative analyses of RISK and enteroid metabolic models

**Figure 4:**
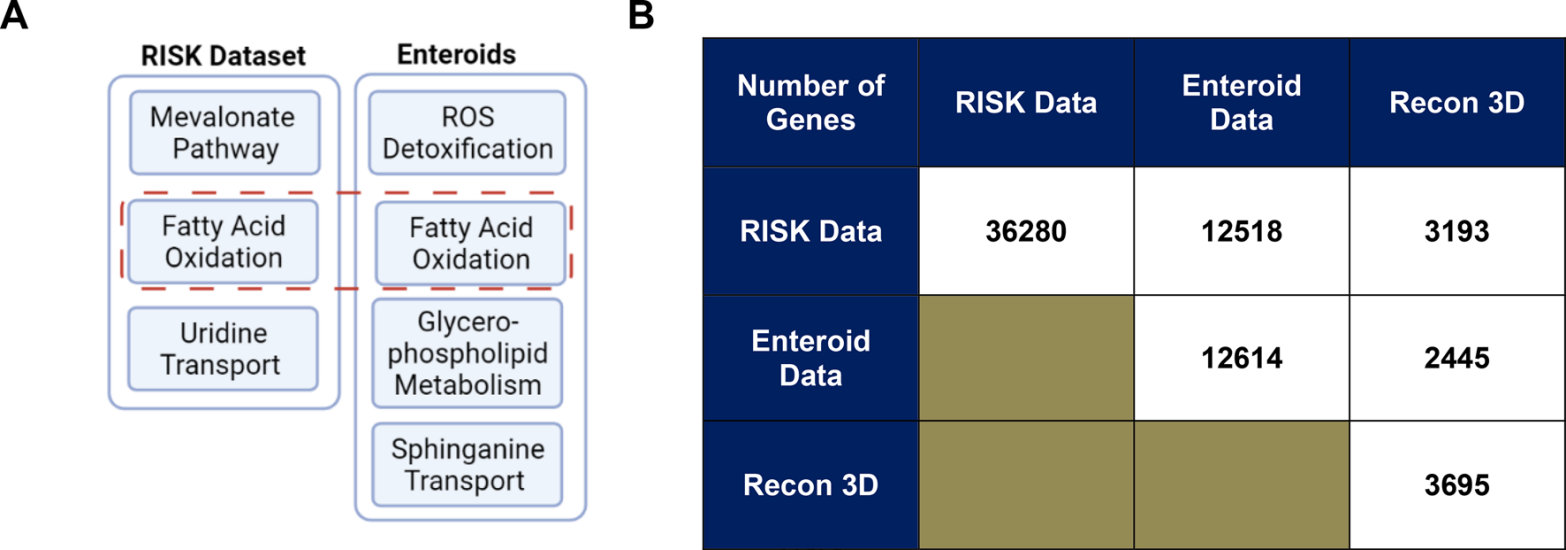
Overlap between gene count data and pathways with an altered flow in the RISK dataset and enteroids: (**A**) Overlap between the reaction "families" that were found to be altered in the RISK dataset and enteroids. After conducting in silico RIPTiDe studies and performing random forest analysis, reactions involved in fatty acid oxidation were found to be altered in both the RISK dataset and the enteroids as detailed in Table 1. (**B**). Gene overlap between the RISK cohort and the ileal organoids- "enteroids." Gene count analysis showed that 99% of the genes present in enteroids were also present in the RISK dataset. Only 96 genes were found to be unique to the enteroids.

Using the computational approaches detailed above, we generated metabolic models of CD in both RISK-derived tissue and enteroids. When these models were compared, we noted that fatty acid metabolism (Fig. 4A) was a common biological process highlighted between RISK and enteroid models, with additional reactions identified in each model. Furthermore, we noted a significant overlap in gene expression between ileal organoids and tissue biopsies (Fig. 4B).

These data indicated that the fatty acid metabolic profile in the tissue sufficiently maps to epithelial cell function. In addition, the gene count data shown in Fig. 4B suggests transcriptomic changes associated with CD may differ based on different specific cell types being analyzed. The difference in gene count between RISK and enteroid data prior to overlaying on Recon3D suggests that the total gene count data in the publicly available transcriptomic datasets included both protein-coding and noncoding mRNA sequencing. By subtracting the enteroid epithelial gene count data (n = 2445 genes), after overlaying it on Recon3D from the gene count data from RISK (n = 3193 genes), we inferred that 748 genes are likely to be related to functions outside of the epithelium that are not represented in enteroids but would be present in an ileal biopsy.

### Contextualization of in silico metabolic modeling approach with metabolomics

**Figure 5:**
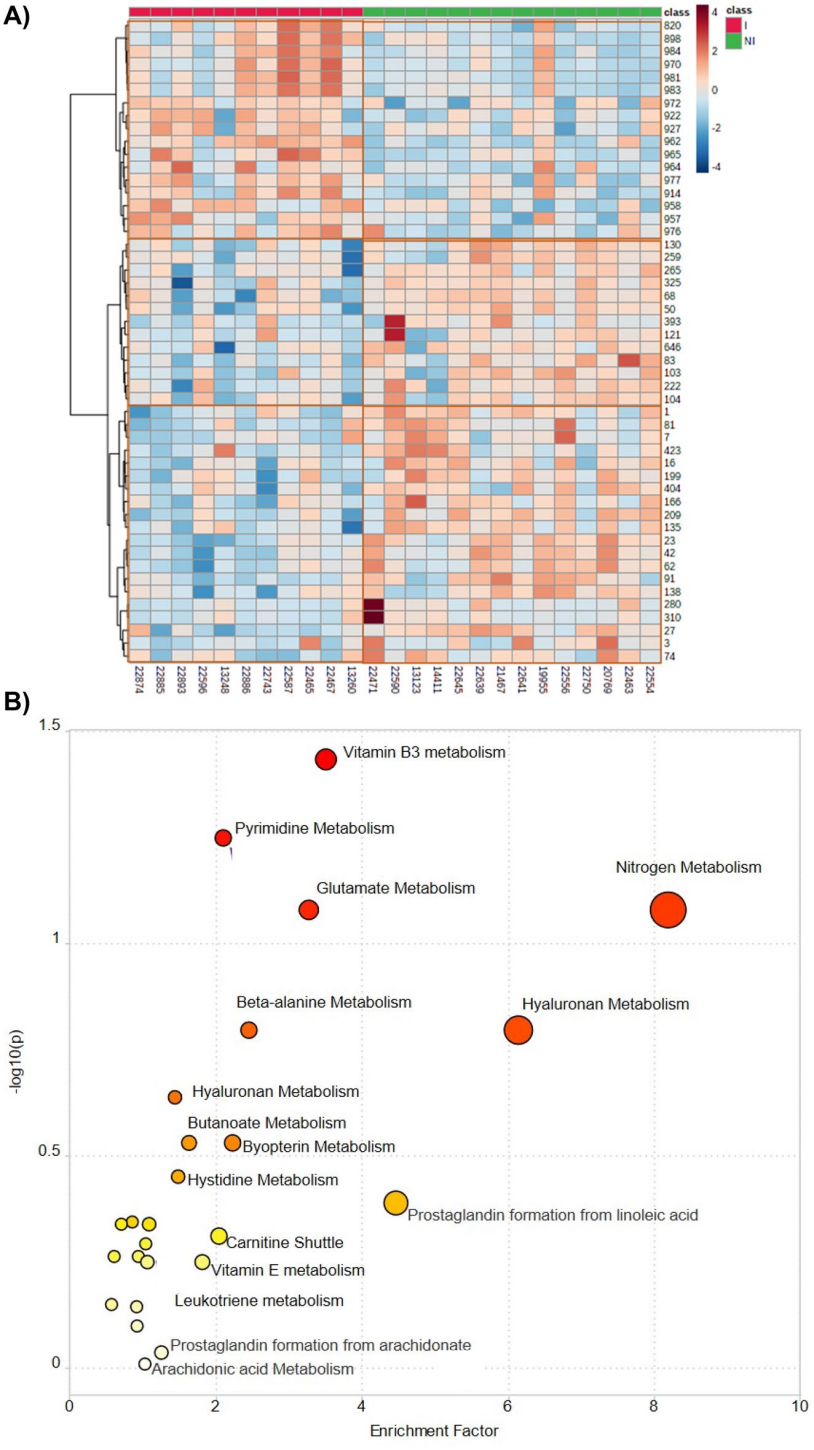
Ileal tissue from inflamed pediatric CD patients vs. non-inflamed have distinct metabolic signatures. (**A**) A dendrogram depicting relative abundances of the 50 most significant compounds (top to bottom) compared between inflamed ileal tissue (red, left) and non-inflamed ileal tissue (green, right) patients (left to right). Data was obtained with reverse phase liquid chromatography mass spectrometry (LC–MS) in positive ion mode. Detailed LC–MS compound information is detailed in Supplemental Table 2. Supplemental Table 2 clarifies the y-axis labels, which are the feature numbers, the compounds, their elemental formulas, their molecular weight, as their chromatographic retention time. (**B**) Pathway analysis of pilot data using the *mummichog* analytic approach showed the enrichment of multiple metabolites including vitamin B3 metabolism, pyrimidine metabolism, nitrogen metabolism and other lipid and amino acid pathways in CD compared to control tissue groups, in alignment with our in silico findings.

**Figure 6:**
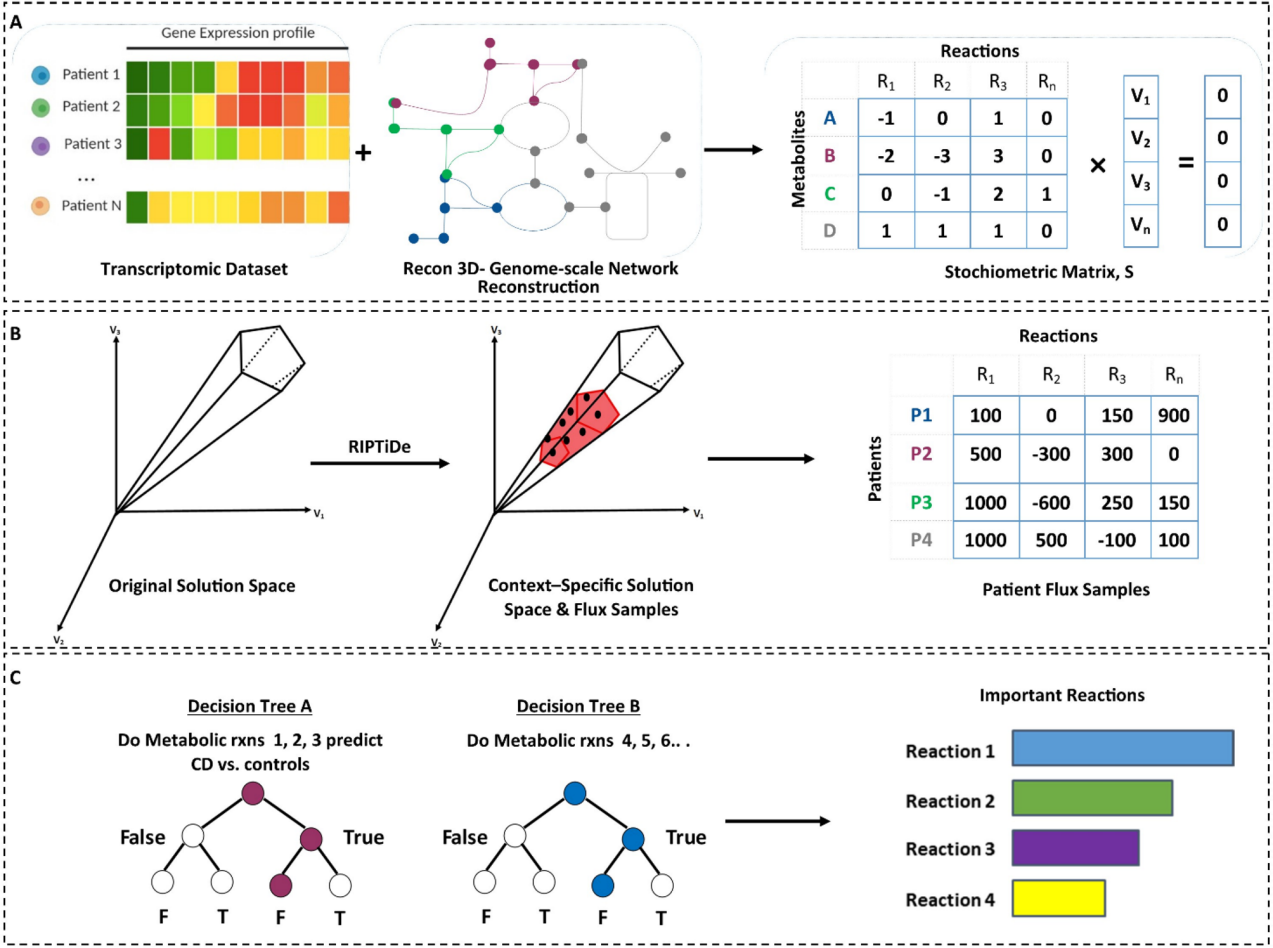
Overview of Methods Framework: (**A**) Risk Stratification and Identification of Immunogenetic and Microbial Markers of Rapid Disease Progression in Children with Crohn’s Disease (RISK) is a prospective inception cohort study, which enrolled 1,276 pediatric patients with IBD at diagnosis at 28 sites in North America between 2008 and 2012. It includes n = 163 patients with ileal Crohn's disease and n = 42 healthy controls. These transcriptomic abundances were overlaid onto Recon3D. Recon 3D is a comprehensive human metabolic network model consisting of three-dimensional (3D) metabolite and protein structure data. (**B**) Flux Balance Analysis workflow. Reaction Inclusion by Parsimony and Transcript Distribution (RIPTiDe) Flux Balance Analysis algorithm was used to generate flux values for the flow of metabolites through our ileal-specific metabolic network reconstruction. RIPTiDe generates stoichiometric equations for reactions and utilizes the transcriptomic expression of critical enzymes and metabolites to quantify flux within a specific disease state. A growth constraint is built into this algorithm to identify the reactions most efficient in generating biomass. **(C**) Random Forest Classifier Overview. Discriminating reactions between CD patient subtypes were determined using Random Forests (RF). The RF classifier used multiple classification trees to sample a given dataset and asked a series of T/F questions related to flux values to "learn" which metabolic reaction fluxes distinguished Crohn's disease versus normal tissues.

As noted above, inflamed pediatric CD patient ileal tissue displays a distinct metabolic signature compared with non-inflamed tissue. Subsequently, we sought to experimentally confirm whether the CD ileum contained metabolic changes versus controls using a small external cohort. To this end, we used mass spectrometry-based lipidomics in an experiment complementary to our in silico metabolic modeling to characterize whether the changes in metabolic pathways correlate to specific changes in metabolite composition. A heat map depicting relative metabolite quantification of the most variable compounds between inflamed (red) and non-inflamed tissue (green) shows clusters of metabolites that demonstrate a distinct metabolic signature of CD compared to control samples (Fig. 5A). Detailed LC–MS compound information can be found in Supplemental Table 2. This table includes the feature numbers for the compounds that make up the y-axis labels of Fig. 6A and the elemental formulas, molecular weights, and chromatographic retention time of the identified lipid species. Significant variations in metabolic alterations within individuals were observed (Fig. 6A), suggesting significant genetic heterogeneity within CD pathogenesis. In addition, we performed pathway analysis using the mummichog analytic approach for untargeted metabolomics, which leverages the metabolic networks KEGG, UCSD Recon1, and the Edinburgh human metabolic network, to predict biological activity without the need for the identification of specific metabolites beforehand. Using this approach, we identified vitamin B3 metabolism, pyrimidine metabolism, nitrogen metabolism, and other lipid and amino acid pathways to be enriched in CD compared to control tissue, in alignment with our in silico predictions and tissue LC–MS measurements (Fig. 5B)^31^.

Together, these findings demonstrate the utility of computational metabolic modeling to specify the characteristic, potentially functional profiles of CD compared to control tissue. Furthermore, the individual metabolic pathways identified by our in silico model displayed significant overlap with the findings from our in vitro enteroid model and our method using untargeted metabolomics, demonstrating the potential value of this metabolic modeling approach. Our work lays the groundwork for future studies to continue probing alterations in the mucosal transcriptome and tissue metabolome for further insights into CD.

## Discussion

This work presents a framework for the in silico metabolic modeling of Crohn's disease (CD) to identify metabolic pathway alterations using archival transcriptomic data and in vitro enteroid models. Cell-to-cell metabolic variability occurs within cell populations as these populations execute a diverse range of functions, which change depending upon environmental impacts on transcriptomic and epigenetic regulation. Metabolic modeling provides a functional snapshot of the ongoing biological processes in a given cell or organism based on complex genotype-phenotype-biochemical relationships. Identifying specific transcriptional signatures of disease through bulk RNA sequencing poses a significant challenge given the heterogeneity in gene expression between patients with the same disease. To distill large amounts of transcriptomic data into a contextualized disease model capable of elucidating alterations in metabolic pathways, which may serve as either disease biomarkers or targets for therapeutic intervention, we have employed metabolic modeling to distinguish alterations in metabolism in diseased versus control tissue across two different datasets. Alterations in metabolic flux can be validated and subsequently quantified externally through in vitro models such as enteroids, untargeted metabolomics, and lipidomics to clarify actionable biomarkers or therapeutic targets. To develop an in silico model, we applied our metabolic modeling approach to the archival RISK dataset, which showed that mevalonate metabolism, fatty acid oxidation, and uridine metabolism have variable flux when comparing patients with CD to controls (Fig. 2), suggesting the relevance of these three classes of biochemical pathways in CD pathogenesis. Our in vitro model comprised organoids generated from patients with CD and controls ileal tissue samples. These organoids were previously found to have a similar transcriptomic signature as in vivo epithelium and retain disease-specific gene expression patterns^28^. In enteroids, we found glycerophospholipid, linoleic acid, and sphingolipid metabolism to be altered (Fig. 3). Lastly, we piloted an additional validation step by using untargeted metabolomics to reveal individual metabolic pathways associated with CD, in which we found vitamin B3, pyrimidine, and glutamate metabolism to be significantly enriched in CD.

The metabolic modeling pipeline detailed in this study showed that reactions involved in mevalonate metabolism were among the top reactions that differentiated CD from controls in the RISK dataset (Fig. 2B). Alterations in mevalonate metabolism in T-lymphocytes have been implicated in decreased inflammatory suppressive activity^31^. In addition, derivatives of mevalonate are known to be directly involved in limiting the cytotoxic effector response of T-lymphocytes in inflammation and the production of immunomodulatory precursors^32^. Alterations of this pathway, such as mevalonate kinase deficiency (MKD), lead to inflammatory bowel disease, or IBD-like intestinal inflammation, possibly due to decreased immunosuppressive isoprenoid intermediates formed through the mevalonate kinase pathway^33^. Therefore, the changes in mevalonate metabolism between CD and controls tissue identified here may contribute to establishing the inflammatory environment associated with CD.

Fatty acid metabolism is a highly regulated process in which dysregulation can lead to an imbalance of pro- and anti-inflammatory mediators. Of note, the dysregulation of fatty acid metabolism contributes to both the type and degree of inflammatory responses in the intestine during IBD^34–36^. Recently, in-depth gene array analysis has also revealed that various genes involved in fatty acid uptake and synthesis are differentially expressed in the ileum of IBD patients^37^. One such pathway differently expressed in IBD patients involves uridine transport and exchange. Uridine is a critical regulator of lipid metabolism and a pathway identified in our current study (Fig. 2D)^38^. As such, pharmacologic agents targeting uridine metabolism are promising candidates for future treatment of inflammatory bowel disease (IBD). Remarkably, in studies utilizing uridine to treat mice with Dextran Sulfate Sodium (DSS)-Induced Colitis, levels of pro-inflammatory cytokines IL-6, IL-1β, and TNF in the serum, and mRNA expression in the colon were significantly decreased in the uridine-treated groups^39^, further demonstrating the critical role for fatty acid metabolism in regulating gastrointestinal inflammation.

In previous studies using cultured ileal organoids (enteroids), no morphological characteristics were observed that could be used to differentiate between CD and controls. Further, when gene expression profiles from enteroids were compared to profiles from freshly isolated intestinal crypts, a strong positive correlation was observed in the mean expression levels of many genes^28,40^. This result suggests that most genes expressed in vivo in the epithelium are also expressed in organoid cultures, demonstrating the immense value of enteroids as an important tool for IBD research. As with the RISK dataset, we grouped altered metabolic reactions identified in organoids based on broad biochemical pathways. We noted that four of the top ten reactions were involved in the dysregulation of fatty acid and phospholipid metabolism (Fig. 3B, C). These findings agree with previous lipid profiling studies of CD patients compared to controls. In these studies, fatty acid and phospholipid metabolism was significantly altered, with most alterations found in glycerophospholipid, linoleic acid, and sphingolipid metabolism pathways^41^. Various metabolites involved in these pathways play a role in inflammation, intracellular signaling, pain, immune function, reproduction, and appetite, perpetuating colitis when dysregulated^42^. Future work will test whether drugs targeting these pathways can alleviate the characteristic inflammation of IBD in patient-derived enteroids, laying the groundwork for further studies into potential therapeutic use^40^. Additionally, the observed alterations of different metabolites of fatty acid oxidation between tissue and organoids suggest mitochondrial dysfunction^43^. Fatty acid biosynthesis has previously been shown to be part of a mitochondrial-to-cytosolic stress response that results when mitochondrial protein synthesis is disrupted^44^. Staining our patient-derived organoids with a mitochondrial membrane potential dye may further help clarify this relationship.

Another biological process that was found to be significantly altered in enteroids derived from CD patients was sphinganine transport (Fig. 3D). Sphinganine is biosynthesized from serine and palmitoyl-CoA through decarboxylating condensation, resulting in a keto intermediate, which is reduced by NADPH. It is then further acylated, then dehydrogenated to form ceramide^45^. Ceramide decreases the release of tumor necrosis factor (TNF), most likely via post-translational regulatory mechanisms and the modulation of TNF-converting enzyme activity^46^. Furthermore, ceramides play an essential role in regulating autophagy, a process strongly linked to the pathogenesis of CD^47^. Sphinganine also blocks lysosomal cholesterol transport and has been linked to Niemann-Pick Type C disease, which predisposes to early-onset IBD with CD phenotype and granuloma formation^48^. However, the exact mechanism by which sphinganine contributes to CD remains unclear and will require further study.

In addition, oxidative stress, caused by increased ROS production, is present locally and systemically in patients with CD^49^. Here, we identified two reactions in this pathway: superoxide dismutase and catalase (Fig. 3A). These enzymes play a role in endogenous antioxidant mechanisms and counteract the effects of excess ROS^50,51^. Notably, multiple enzymes catalyze the production of ROS. Among these enzymes, mucosal NOXs and dual oxidase 2 (DUOX2) have been reported as novel risk factors for IBD^52,53^, further demonstrating that an imbalance in redox homeostasis contributes to the pathogenesis of IBD. Interestingly, In the RISK study, the authors highlight the role of oxidative stress in gut inflammation. Their works detail the enrichment of pro-inflammatory genes, including antimicrobial dual oxidase (DUOX2), and the decreased expression of anti-inflammatory and antioxidant genes, such as those resulting in the production of apoprotein A1 (APOA1) in the ileal tissue of patients with CD^27^.

Using mass spectrometry-based lipidomics, we aimed to pilot an additional level of validation complementary to our in silico metabolic modeling approach. The significant variations in metabolic alterations between diseased and not diseased individuals and within different diseased individuals provide a rationale for future metabolomic analyses of large cohorts examining phenotypic variations within patients with CD. Our metabolomics analysis showed overlaps with our in silico and in vitro modeling. For example, vitamin B3 was found to be significantly enriched, iterating the theme of the dysregulation of lipid metabolism present in CD. Interestingly, high-dose vitamin B3 treatment has been shown to ameliorate ulcerative colitis through increased prostaglandin D2 synthesis in mice^54^. Thus, niacin supplementation can be a potential therapeutic target to be investigated in CD as well. In addition, untargeted metabolomics showed the dysregulation of pathways related to pyrimidine, glutamate, and nitrogen metabolism. Similarly, in the RISK dataset, we found uridine transport and metabolism to be altered between patients with CD and controls, further warranting assessment of the potential efficacy of uridine in CD management.

Limitations in this study are shown in the discrepancy in the specific metabolic reactions that have altered flux when comparing the RISK dataset to the enteroid dataset presents a visible limitation to our study. The use of publicly available, pre-normalized data that does not include row counts presents a limitation to using other normalization techniques for inter-sample comparison. We used Recon3D, which is a generic metabolic network reconstruction and not tissue-specific. In future studies, we hope to generate a tissue-specific reconstruction analogous to the heart-specific iCardio^17^. Further, the inclusion of medium information specific to Crohn’s disease may improve FBA performance, as well as inclusion of detailed information on macromolecular synthesis rates in Crohn’s disease to support or negate assumptions of hypermetabolism . From a biological standpoint, variations in the mean age and ethnicity of patients also have varying effects on the metabolome and lipidome of the enteroids and, thus are confounders in our analysis. In addition, there is an incongruity between the sizes of the two cohorts, as enteroids were generated from a smaller population (Table 1). Furthermore, it is crucial to recognize that enteroids only consist of epithelial tissue, while ileal tissue contains other mucosal elements, including the submucosa, muscularis mucosa, and the immune cells that are present in these layers.CD affects the entire thickness of the epithelium, involving a complicated interaction between genetic factors, growth factors, cytokines, environmental factors such as diet and smoking, and the constitution of the gut microbiome^27^. Thus, not only does the absence of other cell types present a limitation to our analysis, but the absence of an accounting of the gut microbiome signaling in the enteroids is also a shortcoming. In particular, the absent gut microbiome may serve as an explanation for the dissimilarities between altered fatty acid pathways in the RISK dataset and enteroid models^55,56^. In emerging work studying organoids' interaction with other elements of the gastrointestinal system, researchers added microbiome elements to characterize the carcinogenic effects of *E*. coli on intestinal organoids^57^. This work clearly demonstrated that microbial elements may significantly impact the genetic signature of organoids, revealing a technical limitation in using organoids. To address this limitation, other groups have started developing novel organoid culture systems having added resident innate immune cells and fibroblasts and observed their interactions with tissue to enable further use of enteroid as an in vitro model for changes to the gut epithelium^58^. Future studies with these complex organoid systems will allow us to better probe the relationship of the microbiome to the metabolic pathways underlying CD pathogenesis.

The notable strength of this study is the detailed framework for in silico prediction with in vitro validation, pruning genes and metabolic reactions from transcriptomic data, and lipidomics analysis to provide a high-order understanding of alterations in metabolism for specific disease states. These altered pathways may define measurable biomarkers specific to certain disease states or even represent targetable therapeutic options. In this study, we identified metabolic pathways with altered flow in both archival data and enteroid models of Crohn's disease and revealed overlap in the overall biochemical processes in which they occur. In the archival RISK dataset, we found mevalonate, fatty acid, and uridine metabolism to be altered, while in enteroids, we found glycerophospholipid, linoleic acid, and sphingolipid metabolism to be altered (Figs. 2, 3). Thus, fatty acid metabolic pathways offer promising therapeutic targets in managing CD. Due to the overlap between metabolic alterations observed in both enteroids and the RISK datasets, ileal enteroids can offer us insight into epithelial response to various pharmaceutical interventions. In addition, the other metabolic pathways identified by metabolic modeling offer a wide range of targetable metabolites and reactions that can be investigated further. Metabolomics analyses can also be used to further validate these findings by confirming whether the metabolites of the pathways with altered flow are present and measurable in ileal enteroids and biopsies. Future studies will determine whether the metabolites identified in this study can be used to monitor disease progression and treatment response and whether therapeutic targeting of these pathways may provide an effective approach to treating Crohn's disease.

## Methods

### Publicly available transcriptomic data from the RISK inception cohort

RNA sequencing data was obtained from the RISK pediatric prospective inception cohort study^1,27^. All CD patients were required to undergo a baseline colonoscopy during which biopsies were taken to confirm chronic active colitis or ileitis on histology prior to intervention and were followed at a time point 22 months after diagnosis to monitor disease progression^1,27^. Transcriptomic data normalized to reads per kilobase per million (RPKM) transcriptomic data from the RISK study was accessed using the GEO accession series GSE57945. Our analysis included n = 163 patients with ileal CD and n = 42 controls who were not found to have CD on histology.

### In silico metabolic modeling platform applied in vitro on enteroids

Enteroids were derived from a previously established CD cohort (n = 16 CD, n = 12 non-IBD controls)^28^. Publicly available enteroid RNA-seq data was accessed using the accession key PRJNA643576. Data was analyzed using the analytical pipeline designed for the RISK dataset outlined below. RNA sequences from the enteroid study were normalized to RPKM and used for downstream *in-silico* metabolic modeling to validate our i*n-silico* approach using a tissue model of CD.

### In silico identification of relevant metabolic reactions

The publicly available RISK transcriptomic dataset and enteroid transcriptomic data were overlaid onto Recon3D, a human metabolic reconstruction^18^. As RNA sequencing was done on ileal biopsies of patients with Crohn’s disease and controls, by overlaying the gene count data onto Recon3D we obtain an ileum-specific model for this study. Genes present in both the Recon3D network and transcriptomic data were retained, while the rest were removed. Retained reactions were specifically linked with transcriptomically-abundant RISK and enteroid genes. Constraint-Based Reconstruction and Analysis (COBRA) Toolbox was used to remove reactions determined to be inactive and generate an ileal-specific metabolic network reconstruction to contextualize our downstream analyses^59,60^.

### Identifying reactions with differential utilization of relevant metabolic pathways

RIPTiDe, a subset of parsimonious Flux Balance Analysis (pFBA), was used to prune the list of genes and reactions obtained from the diseased and control patient sets^24^.The RISK transcriptomic dataset or enteroid transcriptomic data with Recon3D was used to create a contextualized metabolic model for each patient based on the parsimonious usage of reactions defined by their associated transcriptomic analysis. Multiple possible flux values can be generated from the permissible range of flux bounds for all the active reactions in each patient. The RIPTiDe package randomly samples 50 to 500 flux values depending on the permissible flux ranges. The parameters for the implementation of the RIPTiDe package were kept at default, with the exception of the minimum percent of optimal objective value during the flux balance analysis which we increased from 0.8 to 1 to control the variability of flux ranges assuming a hypermetabolic state in CD^61–63^ . A random flux sampling method was used to generate multiple data points for training the machine-learning random forest model. The scikit learn implementation was used for random forest^20–22,24,25,64,65^. We kept the open_exchange parameter in the COBRA optimizer as “false” to allow the FBA module to automatically determine the maximum and minimum bound of reactions based on transcriptome contextualization and FBA optimization.

### Extracting discriminative reactions using machine learning

Many reactions identified by FBA are essential for normal tissue function and, thus, were found to have similar flow between patients with CD and controls. Therefore, we employed RIPTiDe to extract metabolic reactions that had altered flow between patients with CD and controls. A random forest classifier was used to classify CD versus control patients using reactions found to be altered in the diseased state. An 80–20% ratio was used for separating patient data points to create training data and test data for machine learning modeling. All flux points associated with patients in the training set were used to train the model, and the model was evaluated on flux data points associated with validation patients. Due to the small size of control patients in the RISK and enteroid datasets, a repeated validation approach was used to identify the top reactions in an unbiased approach. Multiple splits of train-validation patients were created to train and evaluate the model. This extensive cross-validation allowed us to demonstrate performance gains and takeaways across the dataset. Top reactions were extracted and aggregated from splits where the model had a high detection accuracy. Due to an imbalance between the control and CD patients, a random under-sampling method was used in each split to balance the number of controls and CD patients. A schematic overview of the methods used can be viewed in Fig. 6.

### Grouping reactions to derive functional groups

The top 20 reactions identified by our metabolic modeling pipeline (detailed above) were extracted from the splits with an F1 score (the harmonic mean of precision and recall) greater than 70% and used for further analysis. This step ensured only reactions which were substantially altered between CD and control patients in the training and validation patients were included in the functional group analysis. Differences between patients with CD and controls were calculated using the Mann–Whitney U test, and a *p* value < 0.05 was considered significant. These top 20 reactions were further grouped based on their metabolic subsystems and description of their overall function based on the virtual metabolic human (VMH) reaction database of Recon3D^18,30^.

### Mass spectrometry-based lipidomics

Ileal tissue samples from pediatric CD patients (n = 11) and control non-inflamed ileal samples (n = 14) were analyzed via non-targeted LC–MS lipidomics assays to semi-quantitatively measure thousands of non-polar compounds, including lipids from the eight main lipid classes (fatty acyls, glycerolipids, glycerophospholipids, sphingolipids, sterol lipids, prenol lipids, saccharolipids, and polyketides) in these complex biosamples. Biopsies were cryopulverized prior to mixing with extraction solvent (IPA) to extract the non-polar lipids while minimizing highly polar compounds and proteins, which would suppress the signals of the desired analytes. The supernatant resulting from the extraction was stored at 4 °C until analysis. A pooled quality control sample was injected at regular intervals (every 5 samples) throughout the sample batch, bracketing the actual samples, which were randomized. Data collection was performed on a ThermoFisher Scientific Q-Exactive HF (QE HF) Hybrid Quadrupole-Orbitrap MS system coupled with a Vanquish Horizon LC system in positive and negative ion modes. Compounds were separated using a ThermoFisher Scientific Accurcore C30 (150 × 2.1 mm, 2.6 µm particle size). The chromatographic method for sample analysis involved elution with 80:20 water:MeCN with 10 mM ammonium formate and 0.1% formic acid (mobile phase A) and MeCN and 0.1% formic acid (mobile phase B) using the following gradient program: 0 min 5% A; 0.5 min 5% A; 8 min 60% A; 10.4 min 60% A; 10.5 min 5% A; 14 min 5% A. The flow rate was set at 0.4 mL/min. The column temperature was set to 40 °C, and the injection volume was 2 µL. Full MS data was acquired with 240,000 resolutions over the 150–2000 m*/z* range. Data processing steps for LC–MS data were carried out using Compound Discoverer v3.0. Compound annotation was carried out based on accurate mass, retention time, isotopic pattern, and MS^2^ fragmentation pattern matching local and public libraries. Annotations were consistent with the current naming convention in the Metabolomics Workbench and reflected the extent of structural information contained in the collected data^66^.

### Ethical approval

All experiments and methods were performed in accordance with the relevant guidelines and regulations at Emory University, including approval by Emory University under IRB MOD004-IRB00085516. All patient samples were collected with informed consent.

## Supplementary Information

Supplementary Information.

## Data Availability

RNA sequencing data for the RISK dataset was accessed on the NCBI Gene Expression Omnibus using the accession code GSE57945 (https://www.ncbi.nlm.nih.gov/geo/query/acc.cgi?acc=GSE57945). RNA sequencing data for the enteroid dataset was accessed at the National Library of Medicine using the accession code PRJNA643576 (https://www.ncbi.nlm.nih.gov/bioproject/?term=643576). The methods of this paper can be accessed at https://github.com/GutIntelligenceLab/ContextualizedMetabolicModel.
