# Supplementary material for: Identifying metabolic shifts in Crohn's disease using 'omics-driven contextualized computational metabolic network models
Source: Sci Rep. 2023 Jan 5;13:203. doi: 10.1038/s41598-022-26816-5 (PMC9814625; doi:10.1038/s41598-022-26816-5)
Supplement: Supplementary file 1 — Supplementary Information. [file 41598_2022_26816_MOESM1_ESM.docx]

| SUPPLEMENTAL TABLE 1: REACTION DETAILS OF THE TOP TEN REACTIONS THAT VARY BETWEEN CD AND CONTROLS | | | |
| --- | --- | --- | --- |
| Reaction ID | Reaction | System/Subsystem | Description |
| 1. Top 10 Reactions Altered in RISK Dataset | | | |
| ATP2ter | amp[r] + atp[c] <=> amp[c] + atp[r] | Transport, endoplasmic reticular | AMP/ATP Transporter, Endoplasmic Reticulum |
| HMGCOAtm | hmgcoa[c] <=> hmgcoa[m] | Transport, mitochondrial | Hydroxymethylglutaryl Coenzyme A Reversible Mitochondrial Transport |
| MEV_Rt | mev_R[c] <=> mev_R[e] | Transport, extracellular | Transport of (R)-Mevalonate |
| r0488 | coa[c] + mev_R[c] + 2.0 nadp[c] <=> 2.0 h[c] + hmgcoa[c] + 2.0 nadph[c] | Cholesterol metabolism | (R)-Mevalonate:NADP+ Oxidoreductase (Coa Acylating) |
| EX_mev_R[e] | mev_R[e] <=> | Exchange/demand reaction | Exchange of (R)-Mevalonate |
| sink_lnlncacoa[c] | lnlncacoa[c] <=> | Exchange/demand reaction | Alpha-Linolenoyl-CoA metabolism |
| EX_uri[e] | uri[e] <=> | Exchange/demand reaction | Exchange of Uridine |
| r1466 | 4.0 h[r] + lnlncgcoa[r] + 2.0 malcoa[r] + 2.0 nadph[r] + 2.0 o2[r] <=> 4.0 co2[r] + 2.0 coa[r] + dlnlcgcoa[r] + 2.0 h2o[r] + 2.0 nadp[r] | Miscellaneous | Long-Chain-Acyl Coenzyme A Dehydrogenase |
| sink_lnlc[c] | lnlc[c] <=> | Exchange/demand reaction | Linoleic acid metabolism |
| URIt | uri[e] <=> uri[c] | Transport, extracellular | Uridine Faciliated Transport in Cytosol |
| 1. Top 10 Reactions Altered in Enteroid Dataset | | | |
| SPODMm | 2.0 h[m] + 2.0 o2s[m] -> h2o2[m] + o2[m] | ROS detoxification | Superoxide Dismutase |
| CATm | 2.0 h2o2[m] -> 2.0 h2o[m] + o2[m] | ROS detoxification | Catalase |
| RE3121R | CE4819[r] + h[r] + nadph[r] <=> CE4817[r] + nadp[r] | Fatty acid oxidation |  |
| PCHOLP_hs | h2o[c] + pchol_hs[c] -> chol[c] + h[c] + pa_hs[c] | Glycerophospholipid metabolism | Choline Phosphatase |
| PSt3 | ps_hs[e] <=> ps_hs[c] | Transport, extracellular | Transport of Phosphatidylserine |
| SPHGNte | sphgn[e] <=> sphgn[c] | Transport, extracellular | Passive Diffusion of Sphinganine into Extracellular Space |
| RE0577C | arachcoa[c] + h2o[c] <=> arach[c] + coa[c] + h[c] | Fatty acid synthesis | Palmitoyl Coenzyme A Hydrolase |
| EX_HC01610[e] | cpppg1[e] <=> | Exchange/demand reaction | Exchange of Coproporphyrinogen |
| HMR_0692 |  | Exchange/demand reaction |  |
| biomass_reaction |  | Exchange/demand reaction | Generic Human Biomass Reaction |

| SUPPLEMENTAL Table 2: LC-MS Information For 50 Most Significant Compounds In Figure 6A. | | | |
| --- | --- | --- | --- |
| Feature Number | **Elemental Formula** | **Molecular Weight (Neutral Species)** | **Chromatographic Retention Time (min)** |
| 820 | C59 H98 N3 O5 P | 959.7249 | 6.92 |
| 898 | C52 H111 N3 O8 P2 S | 999.7564 | 7.01 |
| 984 | C54 H98 N5 O7 P S | 991.6913 | 6.35 |
| 970 | C64 H92 N3 P S | 965.6753 | 6.23 |
| 981 | C26 H57 N5 O7 P2 | 613.3731 | 4.09 |
| 983 | C52 H100 N5 O6 P S | 953.7145 | 6.76 |
| 972 |  | 779.5806 | 4.98 |
| 922 | C5 H15 N4 O8 P | 290.0616 | 0.79 |
| 927 | C7 H15 O11 P | 306.0358 | 0.81 |
| 962 | C22 H43 N O4 | 385.318 | 1.84 |
| 965 | C23 H39 N O2 | 361.297 | 1.59 |
| 977 |  | 743.5702 | 6.03 |
| 914 | C40 H86 N4 O11 | 798.6306 | 6.27 |
| 958 | C15 H31 N O2 | 257.235 | 1 |
| 957 | C40 H86 N5 O11 P | 843.6093 | 6.5 |
| 976 | C21 H39 N O5 | 385.2818 | 1.11 |
| 130 | C36 H65 N3 O4 | 603.4975 | 6.43 |
| 259 | C42 H86 N2 O3 | 666.6618 | 6.42 |
| 265 | C40 H80 N2 O3 | 636.6145 | 4.68 |
| 325 | C42 H80 O11 P2 | 822.5145 | 4.45 |
| 68 |  | 810.5278 | 4.45 |
| 50 |  | 820.5291 | 4.46 |
| 393 |  | 2003.0122 | 5.71 |
| 121 |  | 999.4896 | 4.84 |
| 646 |  | 708.5073 | 6.89 |
| 83 |  | 836.6 | 5.35 |
| 103 |  | 1644.1701 | 5.7 |
| 222 |  | 1982.9391 | 5.92 |
| 104 |  | 868.5306 | 5.73 |
| 1 | C5 H17 N5 O2 S | 211.1103 | 0.82 |
| 81 | C8 H18 N4 O2 | 202.1424 | 0.78 |
| 7 | C7 H10 N2 O3 | 170.0686 | 0.8 |
| 423 | C37 H73 Cl N4 O2 P2 | 702.4922 | 7.13 |
| 16 | C43 H93 Br N5 O12 P | 981.5766 | 6.64 |
| 199 | C44 H81 N S | 655.6094 | 6.89 |
| 404 | C24 H42 N4 O10 S | 578.261 | 2.86 |
| 166 |  | 918.4908 | 5.84 |
| 209 |  | 904.7278 | 8.35 |
| 135 | C69 H132 N4 O3 S | 1097.002 | 5.46 |
| 23 | C23 H33 P S | 372.2029 | 2.42 |
| 42 | C19 H33 N4 O8 P S | 508.1774 | 2.42 |
| 62 | C26 H28 Cl N2 O4 P | 498.1485 | 2.42 |
| 91 |  | 410.1896 | 2.5 |
| 138 |  | 544.1327 | 2.5 |
| 280 | C58 H104 N2 O5 S | 940.768 | 6.9 |
| 310 | C61 H108 O P2 | 918.7863 | 6.91 |
| 27 | C42 H82 N O8 P | 759.5762 | 11.96 |
| 3 | C65 H121 N3 O12 | 1135.8955 | 8.39 |
| 74 | C20 H32 O5 | 352.2239 | 0.91 |

| SUPPLEMENTAL TABLE 3: TRAIN TEST SPLIT AND CLASSIFIER PERFORMANCE | | | | |
| --- | --- | --- | --- | --- |
|  | **A. Risk Cohort** | | **B. Enteroid Cohort** | |
|  | **Crohn's Disease** | **Controls** | **Crohn's Disease** | **Controls** |
| Total Patients | 163 | 42 | 16 | 12 |
| Riptide samples 50-500 flux values per patient depending on the flux range. | | | | |
| Number of Flux Samples | 21024 | 5795 | 2207 | 1722 |
| Number of Reactions in Flux Output | 318 | 434 | 651 | 651 |
| Combined Flux Output for model training | 26819 x 314 | | 3929 x 651 | |

| supplemental table 4: total pruned reactions in each cohort | |
| --- | --- |
| RISK Cohort Reactions | **Enteroid Reactions** |
| 5AOPtm | 5AOPtm |
| ADK1m | ACACT1rm |
| ADK3 | ADK3 |
| ALATA_L | ADK3m |
| ARTFR12 | ALATA_L |
| ATP2ter | ALDD2y |
| ATPtm | AMY1e |
| CBPPer | ARTFR12 |
| CBPter | ARTFR207 |
| CDIPTr | ARTFR211 |
| CITtbm | ARTFR212 |
| CKc | ASPCTr |
| CLS_hs | ATP2ter |
| CYTDt | ATPtm |
| DAGK_hs | C160CPT1 |
| DAGKn_hs | C160CPT2 |
| DAGt | C161CPT22 |
| DATPtn | C181CPT1 |
| DCK1n | CATm |
| DCTPtn | CATp |
| DGAT | CBPPer |
| DGK2m | CBPter |
| DGNSKm | CDIPTr |
| DGSNtm | CITtbm |
| DGTPtn | CKc |
| DIDPtn | CLPNDCPT1 |
| DITPtn | CLS_hs |
| D_LACt2 | COAtm |
| D_LACtm | CRNCARtp |
| DNDPt11m | CSNAT2m |
| DNDPt9m | CSNATm |
| DRPA | CSNATp |
| DTDPtn | CSNATr |
| DTTPtn | CYTDt |
| DUDPtn | CYTK2n |
| EX_atp[e] | CYTK4 |
| EX_cmp[e] | DAGK_hs |
| EX_creat[e] | DAGKn_hs |
| EX_dag_hs[e] | DAGt |
| EX_h2o2[e] | DATPtn |
| EX_idp[e] | DCIm |
| EX_imp[e] | DCK1n |
| EX_lac_D[e] | DCTPtn |
| EX_o2s[e] | DCYTt |
| EX_pe_hs[e] | DESAT16_2 |
| EX_pglyc_hs[e] | DESAT18_3 |
| EX_ps_hs[e] | DGAT |
| EX_Rtotal[e] | DGK2m |
| EX_thmtp[e] | DGNSKm |
| EX_utp[e] | DGSNtm |
| FACOAL1831 | DGTPtn |
| FACOAL244_1 | DHCRD1 |
| FACOAL245_2 | DIDPtn |
| FAEL183 | DITPtn |
| FUMm | D_LACtm |
| GLBRAN | DLNLCGCPT1 |
| GLCter | DNDPt11m |
| GLDBRAN | DNDPt13m |
| GLGNS1 | DNDPt9m |
| GLPASE1 | DRPA |
| GLUt2m | DTDPtn |
| GLYOXm | DTTPtn |
| GLYtm | DUDPtn |
| H2O2t | EICOSTETCPT1 |
| H2Otn | ELAIDt |
| HMGCOAtm | EX_atp[e] |
| Htr | EX_cmp[e] |
| IDPtn | EX_dag_hs[e] |
| ITPtn | EX_elaid[e] |
| LPS2 | EX_h2o2[e] |
| MALtm | EX_hdcea[e] |
| MCCCrm | EX_hexc[e] |
| MDHm | EX_hpdca[e] |
| MGCHrm | EX_idp[e] |
| MI1PS | EX_imp[e] |
| MMMm | EX_lneldc[e] |
| MMTSADm | EX_nrvnc[e] |
| NDPK10 | EX_ocdca[e] |
| NDPK10n | EX_pe_hs[e] |
| NDPK4n | EX_pglyc_hs[e] |
| NDPK6n | EX_ps_hs[e] |
| NDPK8m | EX_ptdca[e] |
| NDPK9n | EX_Rtotal[e] |
| NMNATm | EX_strch1[e] |
| NTPP9 | EX_tethex3[e] |
| O2St | EX_tetpent3[e] |
| PA_HStn | EX_tetpent6[e] |
| PAIL_HStn | EX_thmtp[e] |
| PAIL45P_HStn | EX_ttdca[e] |
| PEPCKm | EX_utp[e] |
| PEt | FACOAL140i |
| PGLYCt | FACOAL150 |
| PI45PLC | FACOAL170 |
| PIter | FACOAL1821 |
| PItn | FACOAL1822 |
| PSt3 | FACOAL1831 |
| RTOT_3 | FACOAL1832 |
| RTOTALt | FACOAL184 |
| SMS | FACOAL203 |
| SUCCt2m | FACOAL204 |
| SUCD1m | FACOAL2042 |
| SUCOAS1m | FACOAL205 |
| SUCOASm | FACOAL224 |
| THMTPt | FACOAL2251 |
| TYRTAm | FACOAL2252 |
| UMPK4 | FACOAL226 |
| UMPK6 | FACOAL244_1 |
| URAt | FACOAL245_1 |
| URIt | FACOAL245_2 |
| UTPtn | FACOAL260 |
| EX_ahdt[e] | FAEL183 |
| EX_ctp[e] | FAOXC2051843m |
| EX_HC01361[e] | FATP9t |
| EX_HC01609[e] | FOLt2 |
| EX_HC01610[e] | FUMm |
| r0055 | GACMTRc |
| r0139 | GGNG |
| r0149 | GLBRAN |
| r0165 | GLCter |
| r0181 | GLDBRAN |
| r0196 | GLGNS1 |
| r0276 | GLPASE1 |
| r0391 | GLUt2m |
| r0407 | GLYAMDTRc |
| r0408 | GLYOXm |
| r0430 | GLYtm |
| r0431 | H2O2t |
| r0472 | H2O2tp |
| r0474 | H2Otn |
| r0475 | HDCEAtr |
| r0488 | HEXCt |
| r0517 | HMGCOASim |
| r0531 | HMGCOAtm |
| r0655 | HPDCACRNCPT1 |
| r0707 | HPDCACRNCPT2 |
| r0774 | HPDCAt |
| r0885 | HSD17B9r |
| r0892 | ICDHyrm |
| r0941 | IDPtn |
| r0942 | ITPtn |
| r1004 | LNELDCt |
| r1050 | LNLCCPT1 |
| r1116 | LNLNCGCPT1 |
| r1156 | LPS2 |
| r1393 | MALTe |
| r1423 | MALtm |
| r1431 | MCCCrm |
| r1466 | MDHm |
| RE0124C | MGCHrm |
| RE0344C | MI14Ptn |
| RE0456N | MI1PS |
| RE0577C | MMMm |
| RE0578C | MMTSADm |
| RE0579C | NDPK10 |
| RE1447N | NDPK10n |
| RE1448N | NDPK4n |
| RE1530C | NDPK6n |
| RE3238C | NDPK7n |
| RE3239C | NDPK8m |
| RE3245C | NDPK9n |
| RE3446C | NMNATm |
| RE3446M | NMNATr |
| PIt8 | NRVNCCOAtx |
| PIt9 | NTPP9 |
| biomass_reaction | O16G2e |
| C50CPT1 | O2ter |
| DOCOSACTDr | O2tp |
| EX_ivcrn[e] | PA_HStn |
| IVCOAACBP | PAIL_HStn |
| IVCRNe | PAIL45P_HStn |
| G6PDH2c | PCHOLP_hs |
| GNDc | PEPCKm |
| NADtm | PEt |
| PGLc | PGLer |
| RPEc | PGLYCt |
| PEPtr | PI45PLC |
| DCMPtr | PI4PLCn |
| XMPtr | PIter |
| UDPGLCURtr | PItn |
| IMPtr | PSt3 |
| EX_dcmp[e] | PTDCAt |
| EX_pep[e] | PYRt2p |
| EX_xmp[e] | RTOT_3 |
| EX_udpglcur[e] | RTOTALt |
| PCREATte | SMS |
| HMCRNc | SPODMm |
| EX_HC00900[e] | STRDNCCPT1 |
| EX_hmcr[e] | STRDNCCPT2 |
| EX_mev_R[e] | SUCCt2m |
| EX_mi1p_D[e] | SUCCt4_2 |
| HC00900t4 | SUCOAS1m |
| HMCRNt | SUCOASm |
| MEV_Rt | TETHEX3t |
| MI1Pt | TETPENT3t |
| EX_5aop[e] | TETPENT6t |
| DM_mi145p[c] | THMTPt |
| EX_pcreat[e] | THYMDtm |
| sink_coa[c] | TMDK1m |
| sink_crvnc[c] | TMNDNCCPT1 |
| sink_lnlc[c] | TMNDNCCPT2 |
| sink_lnlncacoa[c] | TRDR |
| sink_nad[c] | TTDCAtr |
| sink_tag_hs[c] | UMPK4 |
| sink_thmtp[c] | UMPK6 |
| EX_glcn[e] | URAt |
| ADK1 | UREAt5 |
| ADPT | URIt |
| CO2t | UTPtn |
| ENO | EX_ahdt[e] |
| EX_cytd[e] | EX_ctp[e] |
| EX_dgsn[e] | EX_dtmp[e] |
| EX_o2[e] | EX_dttp[e] |
| EX_uri[e] | EX_HC00250[e] |
| GALU | EX_HC01361[e] |
| GAPD | EX_HC01609[e] |
| GK1 | EX_HC01610[e] |
| IMPD | r0055 |
| NDPK1 | r0139 |
| NDPK4 | r0149 |
| NDPK6 | r0165 |
| NDPK9 | r0173 |
| O2t | r0181 |
| PGI | r0193 |
| PGM | r0196 |
| PGMT | r0245 |
| PIt6b | r0276 |
| PPM | r0280 |
| PRPPS | r0309 |
| RNDR1 | r0391 |
| RNDR4 | r0407 |
| RPI | r0408 |
| SPODM | r0423 |
| TALA | r0425 |
| TKT1 | r0430 |
| TKT2 | r0431 |
| TPI | r0472 |
| UMPK | r0474 |
| URIDK3 | r0475 |
| r0570 | r0488 |
| EX_nac[e] | r0494 |
| CYTK1 | r0497 |
| EX_ura[e] | r0509 |
| GALT | r0517 |
| LGTHL | r0531 |
| PIt7 | r0584 |
| PYK | r0638 |
| TYRTA | r0643 |
| UGLT | r0651 |
| DCMPDA | r0655 |
| PAIL_hs_t1e | r0707 |
| GLCNte | r0774 |
| SPHGNte | r0797 |
| EX_sphgn[e] | r0801 |
| 4GLU56DIHDINDt | r0821 |
| EX_4glu56dihdind[e] | r0885 |
| NH4tr | r0892 |
| EX_pail_hs[e] | r0940 |
| HMR_0192 | r0941 |
| HMR_0204 | r0983 |
| HMR_0209 | r0984 |
| HMR_0233 | r1004 |
| HMR_0241 | r1050 |
| HMR_0245 | r1116 |
| HMR_0255 | r1154 |
| HMR_0271 | r1156 |
| HMR_0279 | r1292 |
| HMR_0289 | r1393 |
| HMR_0293 | r1418 |
| HMR_0297 | r1423 |
| HMR_0309 | r1431 |
| HMR_0319 | r1433 |
| HMR_0327 | r1454 |
| HMR_0345 | r1466 |
| HMR_0385 | r2374 |
| HMR_0389 | r2420 |
| HMR_0393 | r2435 |
| HMR_0429 | r2473 |
| HMR_0546 | r2516 |
| HMR_0588 | RE0124C |
| HMR_0600 | RE0344C |
| HMR_0607 | RE0456N |
| HMR_0668 | RE0577C |
| HMR_0683 | RE0578C |
| HMR_0691 | RE0579C |
| HMR_0692 | RE1447N |
| HMR_0753 | RE1448N |
| HMR_1095 | RE1530C |
| HMR_1267 | RE2563C |
| HMR_1284 | RE2640C |
| HMR_2296 | RE2954C |
| HMR_2702 | RE3106R |
| HMR_2851 | RE3110R |
| HMR_2852 | RE3112R |
| HMR_2973 | RE3113R |
| HMR_4266 | RE3119R |
| HMR_6617 | RE3120R |
| HMR_6619 | RE3121R |
| HMR_6781 | RE3122R |
| HMR_7745 | RE3238C |
| HMR_7747 | RE3239C |
| HMR_7748 | RE3245C |
| HMR_7749 | RE3272N |
| HMR_7898 | RE3273C |
| HMR_8510 | RE3432C |
| HMR_8585 | RE3446C |
| HMR_9187 | RE3446M |
| HMR_9674 | PIt8 |
| HMR_5099 | PIt9 |
| EX_M01966[e] | biomass_reaction |
| sink_glu_L[c] | C140CPT1 |
| DM_4glu56dihdind[c] | C30CPT1 |
| ATPS4mi | C50CPT1 |
| NADH2_u10mi | DOCOSACTDr |
|  | EX_doco13ac[e] |
|  | EX_ivcrn[e] |
|  | IVCOAACBP |
|  | IVCRNe |
|  | SUCCACT |
|  | FOLTle |
|  | GLYGLYCNc |
|  | DTMPKm |
|  | G6PDH2c |
|  | GNDc |
|  | NADtm |
|  | PGLc |
|  | RPEc |
|  | CBASPte |
|  | EX_cbasp[e] |
|  | GLYALDtr |
|  | PEPtr |
|  | DCMPtr |
|  | UDPGLCURtr |
|  | IMPtr |
|  | EX_dcmp[e] |
|  | EX_glyald[e] |
|  | EX_pep[e] |
|  | EX_udpglcur[e] |
|  | NACSMCTte |
|  | PCREATte |
|  | LNLCCRNNAt |
|  | EX_HC00900[e] |
|  | EX_lnlccrn[e] |
|  | EX_mev_R[e] |
|  | EX_mi1p_D[e] |
|  | HC00900t4 |
|  | MEV_Rt |
|  | MI1Pt |
|  | EX_5aop[e] |
|  | EX_ddca[e] |
|  | EX_alaarggly[e] |
|  | EX_alaglylys[e] |
|  | EX_argglygly[e] |
|  | ALAARGGLYt |
|  | ALAGLYLYSt |
|  | ARGGLYGLYt |
|  | ALAARGGLYr |
|  | ALAGLYLYSr |
|  | ARGGLYGLYr |
|  | MMALtm |
|  | CE2512te |
|  | DM_mi145p[c] |
|  | DM_mi14p[c] |
|  | DDCAte |
|  | EX_pcreat[e] |
|  | DM_C02712[c] |
|  | sink_Tyr_ggn[c] |
|  | sink_coa[c] |
|  | sink_dgchol[c] |
|  | sink_glygn2[c] |
|  | sink_lnlncacoa[c] |
|  | sink_nad[c] |
|  | sink_tag_hs[c] |
|  | sink_thmtp[c] |
|  | EX_glcn[e] |
|  | ADK1 |
|  | ADPT |
|  | ASPTA |
|  | CO2t |
|  | DURIPP |
|  | ENO |
|  | EX_adn[e] |
|  | EX_cytd[e] |
|  | EX_dcyt[e] |
|  | EX_dgsn[e] |
|  | EX_o2[e] |
|  | EX_orn[e] |
|  | EX_succ[e] |
|  | EX_thymd[e] |
|  | EX_uri[e] |
|  | FBA |
|  | FUM |
|  | GALU |
|  | GAPD |
|  | GHMT2r |
|  | GK1 |
|  | H2Ot |
|  | NDPK1 |
|  | NDPK3 |
|  | NDPK4 |
|  | NDPK5 |
|  | NDPK6 |
|  | NDPK8 |
|  | NDPK9 |
|  | O2t |
|  | PGI |
|  | PGM |
|  | PGMT |
|  | PIt6b |
|  | PPM |
|  | PRPPS |
|  | PUNP1 |
|  | RNDR1 |
|  | RNDR4 |
|  | RPI |
|  | TALA |
|  | TKT1 |
|  | TKT2 |
|  | TPI |
|  | UMPK |
|  | UREAt |
|  | URIDK3 |
|  | r0570 |
|  | EX_nac[e] |
|  | CYTK1 |
|  | EX_ura[e] |
|  | FACOAL161 |
|  | GALT |
|  | LDH_L |
|  | LGTHL |
|  | MDH |
|  | PIt7 |
|  | PYK |
|  | UGLT |
|  | EX_pyr[e] |
|  | PYRt2r |
|  | DCMPDA |
|  | PAIL_hs_t1e |
|  | HDD2CRNte2 |
|  | GLCNte |
|  | EX_hdd2crn[e] |
|  | SPHGNte |
|  | EX_sphgn[e] |
|  | C13856te |
|  | FDPte |
|  | EX_fdp[e] |
|  | 4GLU56DIHDINDt |
|  | EX_4glu56dihdind[e] |
|  | NH4tr |
|  | EX_pail_hs[e] |
|  | HMR_0183 |
|  | HMR_0184 |
|  | HMR_0185 |
|  | HMR_0188 |
|  | HMR_0192 |
|  | HMR_0200 |
|  | HMR_0204 |
|  | HMR_0207 |
|  | HMR_0209 |
|  | HMR_0233 |
|  | HMR_0241 |
|  | HMR_0245 |
|  | HMR_0255 |
|  | HMR_0259 |
|  | HMR_0267 |
|  | HMR_0271 |
|  | HMR_0279 |
|  | HMR_0289 |
|  | HMR_0293 |
|  | HMR_0297 |
|  | HMR_0301 |
|  | HMR_0305 |
|  | HMR_0309 |
|  | HMR_0319 |
|  | HMR_0323 |
|  | HMR_0327 |
|  | HMR_0345 |
|  | HMR_0381 |
|  | HMR_0385 |
|  | HMR_0389 |
|  | HMR_0393 |
|  | HMR_0429 |
|  | HMR_0433 |
|  | HMR_0437 |
|  | HMR_0546 |
|  | HMR_0588 |
|  | HMR_0600 |
|  | HMR_0607 |
|  | HMR_0653 |
|  | HMR_0654 |
|  | HMR_0657 |
|  | HMR_0668 |
|  | HMR_0683 |
|  | HMR_0691 |
|  | HMR_0692 |
|  | HMR_0753 |
|  | HMR_1095 |
|  | HMR_1267 |
|  | HMR_1280 |
|  | HMR_1284 |
|  | HMR_2296 |
|  | HMR_2365 |
|  | HMR_2602 |
|  | HMR_2603 |
|  | HMR_2604 |
|  | HMR_2605 |
|  | HMR_2606 |
|  | HMR_2607 |
|  | HMR_2611 |
|  | HMR_2620 |
|  | HMR_2621 |
|  | HMR_2622 |
|  | HMR_2633 |
|  | HMR_2634 |
|  | HMR_2644 |
|  | HMR_2648 |
|  | HMR_2649 |
|  | HMR_2650 |
|  | HMR_2651 |
|  | HMR_2652 |
|  | HMR_2653 |
|  | HMR_2657 |
|  | HMR_2660 |
|  | HMR_2661 |
|  | HMR_2662 |
|  | HMR_2675 |
|  | HMR_2676 |
|  | HMR_2677 |
|  | HMR_2681 |
|  | HMR_2682 |
|  | HMR_2683 |
|  | HMR_2684 |
|  | HMR_2685 |
|  | HMR_2686 |
|  | HMR_2687 |
|  | HMR_2688 |
|  | HMR_2689 |
|  | HMR_2693 |
|  | HMR_2695 |
|  | HMR_2697 |
|  | HMR_2699 |
|  | HMR_2700 |
|  | HMR_2701 |
|  | HMR_2702 |
|  | HMR_2708 |
|  | HMR_2709 |
|  | HMR_2710 |
|  | HMR_2715 |
|  | HMR_2722 |
|  | HMR_2733 |
|  | HMR_2734 |
|  | HMR_2735 |
|  | HMR_2736 |
|  | HMR_2737 |
|  | HMR_2738 |
|  | HMR_2739 |
|  | HMR_2740 |
|  | HMR_2741 |
|  | HMR_2771 |
|  | HMR_2772 |
|  | HMR_2773 |
|  | HMR_2774 |
|  | HMR_2775 |
|  | HMR_2776 |
|  | HMR_2851 |
|  | HMR_2852 |
|  | HMR_2861 |
|  | HMR_2862 |
|  | HMR_2863 |
|  | HMR_2864 |
|  | HMR_2865 |
|  | HMR_2866 |
|  | HMR_2867 |
|  | HMR_2868 |
|  | HMR_2884 |
|  | HMR_2886 |
|  | HMR_2888 |
|  | HMR_2890 |
|  | HMR_2973 |
|  | HMR_2982 |
|  | HMR_3018 |
|  | HMR_3021 |
|  | HMR_3023 |
|  | HMR_3053 |
|  | HMR_3056 |
|  | HMR_3106 |
|  | HMR_3170 |
|  | HMR_3171 |
|  | HMR_3172 |
|  | HMR_3173 |
|  | HMR_3174 |
|  | HMR_3175 |
|  | HMR_3176 |
|  | HMR_3177 |
|  | HMR_3186 |
|  | HMR_3187 |
|  | HMR_3188 |
|  | HMR_3189 |
|  | HMR_3222 |
|  | HMR_3223 |
|  | HMR_3224 |
|  | HMR_3225 |
|  | HMR_3234 |
|  | HMR_3235 |
|  | HMR_3236 |
|  | HMR_3237 |
|  | HMR_3240 |
|  | HMR_3241 |
|  | HMR_3242 |
|  | HMR_3243 |
|  | HMR_3326 |
|  | HMR_3327 |
|  | HMR_3328 |
|  | HMR_3329 |
|  | HMR_3396 |
|  | HMR_3397 |
|  | HMR_3411 |
|  | HMR_3414 |
|  | HMR_3416 |
|  | HMR_3424 |
|  | HMR_3425 |
|  | HMR_3431 |
|  | HMR_3432 |
|  | HMR_3433 |
|  | HMR_3953 |
|  | HMR_4261 |
|  | HMR_4266 |
|  | HMR_6617 |
|  | HMR_6619 |
|  | HMR_7748 |
|  | HMR_7749 |
|  | HMR_7757 |
|  | HMR_7898 |
|  | HMR_8476 |
|  | HMR_8510 |
|  | HMR_8585 |
|  | HMR_8884 |
|  | HMR_9187 |
|  | HMR_9674 |
|  | CDCA24GSc |
|  | DM_cdca24g[c] |
|  | EX_C13856[e] |
|  | EX_M00117[e] |
|  | EX_M03117[e] |
|  | EX_M01966[e] |
|  | sink_leu_L[c] |
|  | sink_lys_L[c] |
|  | sink_met_L[c] |
|  | DM_4glu56dihdind[c] |
|  | ATPS4mi |
|  | NADH2_u10mi |
|  | CYOOm3i |
